# Landslide risk evaluation method of open-pit mine based on numerical simulation of large deformation of landslide

**DOI:** 10.1038/s41598-023-42736-4

**Published:** 2023-09-16

**Authors:** Lan Jia, Jiaqi Wang, Shisong Gao, Linhao Fang, Dong Wang

**Affiliations:** College of Mining, Liaoning Technical University, Fuxin, 123000 Liaoning China

**Keywords:** Natural hazards, Engineering

## Abstract

It is of great practical significance to carry out quantitative risk assessment of landslide disaster to protect people’s lives and property safety and maintain the sustainable development of social economy. The delineation of landslide disaster range is the key link of landslide risk evaluation in open-pit mine. This study took the open-pit coal mine coal mine in Block I of Thar coal field in Pakistan as the research background, and constructed a framework for landslide risk evaluation in open-pit mines. Based on the numerical simulation method of large deformation of landslide, the disaster range of landslide in open-pit mine was delineated. The stability of stope slope was analyzed based on rigid body limit equilibrium method. The probability of landslide instability was calculated based on Monte Carlo method. And the comprehensive fuzzy evaluation model was established to calculate the total risk value of landslide. The results showed that: through numerical simulation and empirical formula calculation, the landslide disaster range was accurately delineated, and the comprehensive vulnerability of the disaster bearing body was determined to be medium vulnerability; the annual probability of landslide instability under natural conditions was 0.003, which belongs to moderate risk. The annual probability of landslide instability under rainstorm conditions was 0.024, which was highly dangerous; the annual probability of landslide instability under seismic conditions was 0.018, which was highly dangerous. Under natural conditions, the total risk value of landslide was 114,686.4 rmb, and the annual mortality rate of population was 0.0255 people/year. The total risk value of landslide under rainstorm condition was 11,707,570 rmb, and the annual mortality rate of population was 0.18375 people/year. The total risk value of landslide under earthquake condition was 43,007,400 rmb, and the annual mortality rate of population was 0.135 person/year, which was an unacceptable risk. The economic loss was a small geological disaster risk under natural conditions, and it was a medium-sized geological disaster risk under both rainstorm and earthquake conditions. Therefore, landslide prevention and control and management measures such as slope deformation monitoring were proposed to ensure the safety of personnel and property in open-pit mines.

## Introduction

Landslide is a common geological disaster that poses a major threat to people’s production, life and social activities, and is seriously destructive to the surrounding environment. At present, with the acceleration of human industrialization and the intensification of engineering activities, more and more landslides caused by human engineering activities appear, among which the landslide caused by mining is one of them. Open-pit mining engineering changes the balance state of the original ground stress. With the excavation of the open pit, the deformation of the slope rock mass also occurs, and most of the open pit coal mines have appeared on landslides. In view of the prevention and control of landslide disasters in open-pit mines, it is particularly important to scientifically and reasonably predict the harm degree of mine landslides to personnel and economic property, effectively reduce the losses caused by mine landslides, ensure the safe and economic operation of mines, and strengthen the research work of mine landslide disaster risk evaluation.

Landslide disaster risk evaluation has attracted more and more attention, and has gradually become one of the frontier topics in the international research on landslide disasters. Scholars around the world have begun to pay attention to the research work of landslide disaster risk evaluation, and continue to strengthen the theoretical level in the field of landslide disaster research. For the study of quantitative evaluation of landslide risk, such as Cheng^1^, Liu et al.^2^, Luo^3^ calculated the probability of landslide instability under natural, rainfall, earthquake and other conditions based on stability analysis and Monte Carlo method, and the landslide risk evaluation was carried out based on the actual case. Mei et al.^4^ used the material point method to simulate the process of landslide macrodeformation, the slope reliability method to calculate the probability of landslide failure, evaluating landslide vulnerability and consequences based on the results of landslide large deformation simulation, and quantitatively assessed the landslide risk by the product of landslide damage probability, vulnerability and consequences, and proposed a quantitative risk assessment method based on the simulation of large deformation process of landslide. After the twenty-first century, many scholars who study landslide disasters combine GIS with landslide disaster research. For example, Zhang^5^ applied analytic hierarchy process (AHP) to evaluate landslide risk and calculated the weights of each factor, while using GIS methods to obtain the landslide risk assessment grid map in the study area, and the study area was roughly divided into four levels: zero risk, low risk, medium risk and high risk. Li et al.^6^ analyzed the relationship between each risk assessment index and the relative density of landslide disaster points based on GIS technology, and used analytic hierarchy process (AHP) and information model after grading each index to evaluate and regionalize the landslide disaster risk in the study area. In recent years, the quantitative risk assessment method of landslide based on probability theory and numerical simulation have been widely studied^7,8^. This method could take uncertainty factors in geotechnical engineering into quantitative consideration, calculate the probability of landslide occurrence quantitatively, and use numerical simulation to quantitatively assess the consequences of landslide failure. For example, Huang et al.^9^ proposed a framework for landslide risk assessment considering multiple failure modes, that was, applying stochastic limit analysis method to simulate slope failure while using sliding volumes as a consequence, and then conduct quantitative assessment of landslide risk. On the basis of the above, Ali et al.^10^ proposed a quantitative risk assessment framework for rainfall-induced landslides, taking the sliding depth as the failure result to conduct a quantitative assessment of landslide risk. In addition, Li et al.^11^ proposed a regional probability risk analysis method for slope instability, and enhanced the visualization of landslide risk assessment results by drawing regional probability maps.

Landslide instability probability is the main research content of landslide risk evaluation. Li et al.^12^, based on the known development characteristics of Guanyin Mountain landslide, and considering the randomness of physical and mechanical parameters of rock and soil mass, by combining the Bishop method of landslide stability analysis with the Monte Carlo method, evaluated the failure probability of Guanyin Mountain landslide, and discussed the cause mechanism of landslide. Lin^13^ drew the profile of the accumulation body through the large-scale landslide site images in the study area, combined mathematical morphology with Monte Carlo simulation method to extract features such as topography, skeleton and boundary, and then generated a simulation calculation model of the accumulation body instability probability through ANSYS grid to analyze the instability probability of the accumulation body under the influence of rainfall factors. It provided scientific basis for formulating reasonable management measures and preventing the accumulation body instability disaster caused by such factors. Peng^14^ made some modifications to the relationship between the landslide intensity and the ability of the risk units to resist the disasters, and he reduced the values of these two indicators and controlled the evaluation range between 0 and 1. And this model was applied to the risk evaluation of the Three Gorges reservoir area.

It can be seen from the above that the risk evaluation process of landslide disaster is complex. Although scholars have done a lot of research on landslide risk evaluation, most of them are aimed at the risk evaluation of landslide disasters in mountains. Prediction and evaluation of mine landslide disasters risk can help miners better understand the degree and scope of landslide disasters, make targeted prevention and control plans and preparations in advance, and guide the reasonable implementation of major engineering activities. Therefore, it is a necessary and relevant task to conduct landslide risk assessment for mining areas. However, there is still a lack of systematic research on the risk assessment of the slopes of open pit mines, especially the lack of research on the methods of accurately delineating the scope of landslide disasters. Thar coal field in Pakistan is a desert area. With the continuous development of mining process, a multi-weak layer tall soft rock slope has been formed, which leads to the occurrence of localized flaky sides, cracks and landslides. The slope stability problem of this open-pit coal mine is prominent. In this paper, we proposed the landslide risk assessment model of open-pit mines slopes and the delineation method of landslide scope based on numerical simulation and theoretical analysis, taking the open-pit coal mine coal mine in Block I of Thar coal field in Pakistan as an example. It provide reference and guidance for landslide risk assessment of similar open pit mines, and has important practical significance and theoretical value for the prevention and control of landslide disasters and safe production in open-pit mines.

## Engineering geological background of slope

**Figure 1:**
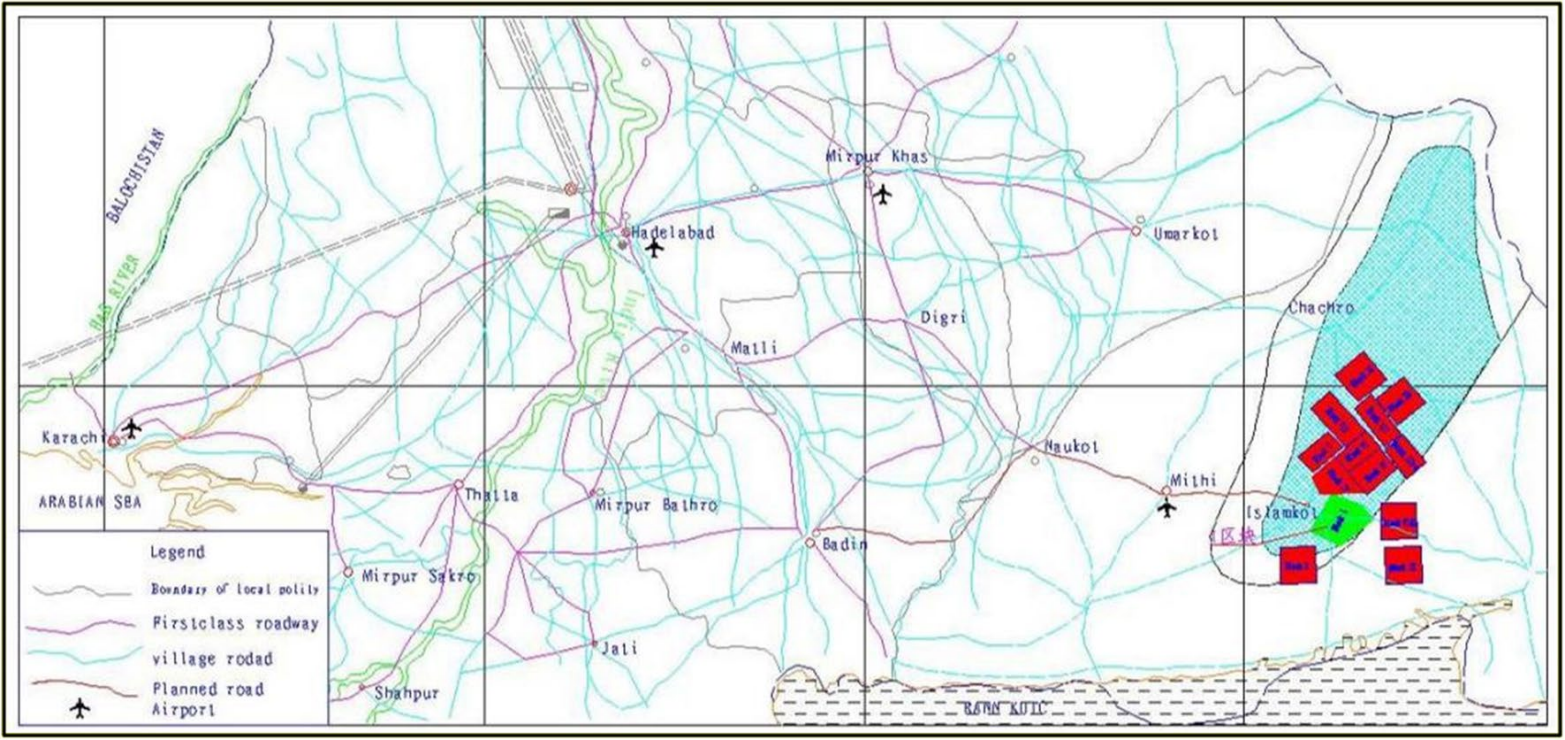
Traffic location map.

The Thar coal field is located in the Thar Desert in the southeast of Sindh province of Pakistan, under the jurisdiction of MIDI City. The geographical coordinates are: East longitude 69° 45′–70° 45′, north latitude 24°15′–24° 45′. The open-pit coal mine in Block I of Thar Coal field in Pakistan is located at the southwestern edge of Thar Desert and is an eolian dune landform. The terrain is undulating low and medium dunes, with flat sand between the dunes. There is no surface water body in the area, the average monthly temperature is 15 ℃–34.7 ℃, the average annual temperature is 25.6 ℃–27.3 ℃, and the extreme maximum temperature is 51 ℃. The regularity of rainfall is not obvious, the main rainfall is concentrated in July and August, the lowest rainfall in November, December and January of the following year, the average rainfall is 248.8 mm. The main wind direction is southwest wind from March to October, northeast wind and northwest wind in December and January, with high wind speed in summer and low wind speed in winter. The monthly average wind speed is 0.31 m/s–10.85 m/s, and the annual average wind speed is 3.20 m/s. Its traffic location was shown in Fig. 1.

**Figure 2:**
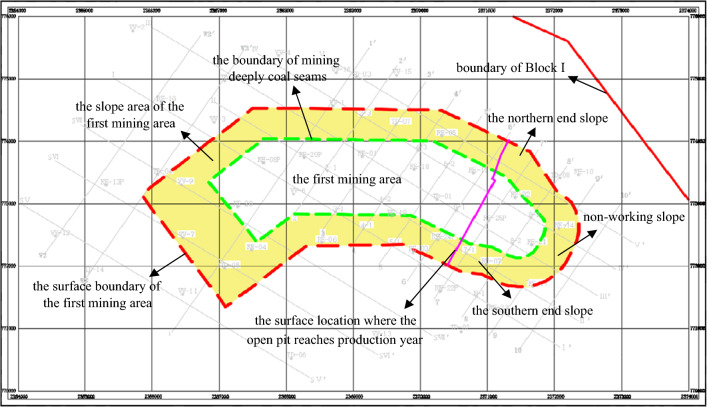
Plan of the first mining area.

**Figure 3:**
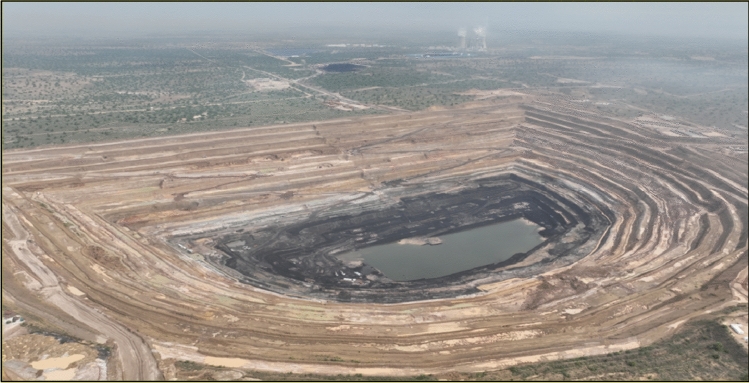
Mining status diagram of the first mining area.

The designed production capacity of the open-pit coal mine coal mine in Block I of Thar coal field in Pakistan is 7.8 million tons/year. The initial pulling ditch is located in the east of the first mining area and advances in both directions to the west and to the east along the strike of the coal seam. The formation dip is gentle and there are no faults in the area. The plan of the first mining area and the mining status diagram were shown in Figs. 2 and 3.

**Figure 4:**
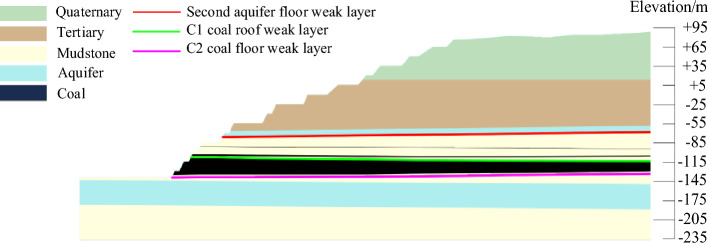
Engineering geological profile of slope.

The main rock (soil) layers within the slopes of the study mine can be divided into three stages: Quaternary aeolian sand layer (I), Neogene Pliocene soft rock layer (II), Paleogene Paleocene-Eocene soft rock layer (III). The Tar coalfield in Pakistan is an extremely wide and gradual syncline along the NNE direction. Layers occurrence gentle, the dip angle is generally about 2°. The undulating coal seam basement leads to slight undulation in the lower part of coal measures strata. Three key weak layers are developed in this area, whose lithology is mainly mudstone, carbonaceous mudstone or clayey siltstone. The mudstone is characterized by high content, loose structure and poor stability. But its water absorption and water holding capacity are strong, and it is easy to soften, expand and disintegrate after encountering water. Three aquifers are developed in the area, all of which are porous aquifers. Among them, the Quaternary sand dune aquifer is a phreatic aquifer. While the Neogene Pliocene bottom sandstone aquifer of the coal seam roof and floor and the Paleogene Paleocene-Eocene bottom sandstone aquifer are confined aquifers. The geological factors such as stratum lithology, weak layers, and the location of aquifer endowment of slopes in the study mine were shown in Fig. 4.

According to the analysis results of geological conditions, the rock mass of the stope slope in this block is low in strength, belonging to soft rock slope, and rich in three-layer aquifers, which is extremely unfavorable to the stability of slope. The bottom of the second-layer aquifers, C1 coal roof and C2 coal floor are the key control weak layers, resulting in increased landslide risk. Therefore, through analysis, it was determined that the main influencing factors of slope stability were lithology of the strata, water, weak structural surfaces and slope form.

According to the control theory of rock mass structure, the failure mode of slope mainly depends on the development of joints and fissures. Slope instability mainly depends on the relationship between anti-sliding force and sliding force^15^. The slope of the open-pit coal mine is mainly controlled by the weak layer of the second aquifer floor in the slope, the weak layer of the C1 coal roof, and the weak layer of the C2 coal floor. The potential landslide mode is analyzed as arc sliding or cutting-bedding sliding with the key weak layer as the bottom interface.

According to the provisions of the slope safety coefficient in the ' Code for design of open pit mine of coal industry ' (GB50197-2015), considered the importance of the stope and the proven degree of the occurrence conditions, the safety reserve coefficient of the stope slope was 1.3. According to the physical and mechanical parameters of the rock and soil mass of the slope selected in the "Preliminary Design Specification of Open-Pit Coal Mine in Block I of Thar Coal Field in Pakistan"," the Hydrogeological Exploration Report of Block I of Thar Coal Field in Pakistan", " and the Overall Report on Slope Stability Analysis of 7.8 million tons/year Open-Pit Coal Mine Project in Block I of Thar Coal Field in Pakistan", the physical and mechanical indexes of rock and soil bodies of each layer selected in this study are determined in Table 1.

**Table 1:** Physical and mechanical indexes of rock-soil mass.

Rock formation	Cohesion C/kPa	Internal friction angle φ/°	Volumetric weight γ/kN m^−3^
Quaternary fine sand	10	33	20
Clay siltstone	110	22	21
first aquifer	71	19	19.56
second aquifer	10	31	21
third aquifer	5	35	19
mudstone	100	18	18
Coal	180	30	12
weak layer	5	18	20

## Establishment of open-pit mine landslide risk evaluation framework

**Figure 5:**
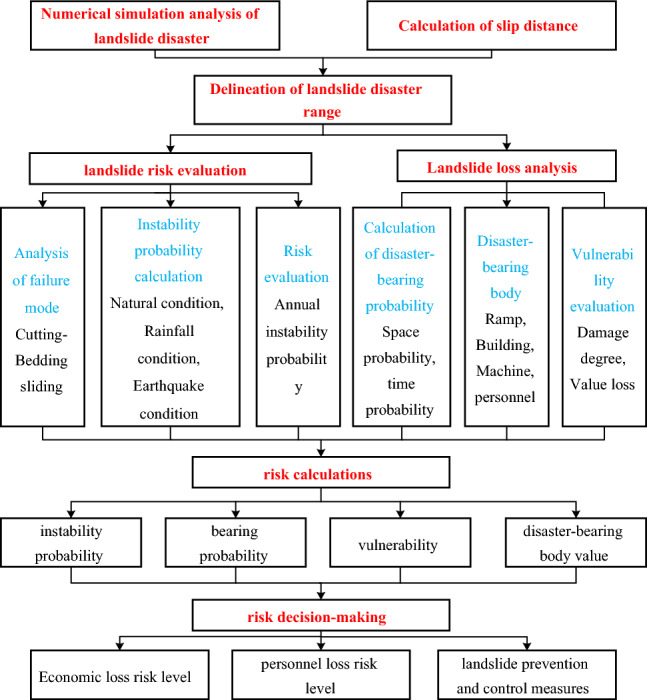
Landslide disaster risk evaluation framework.

The main content of landslide risk evaluation is to analyze the possible losses caused by landslides and provide a theoretical basis for the risk management of landslide disasters. With the wide application of numerical simulation methods, combined with mathematical methods such as probability theory, it is possible to quantify the risk of landslide disasters. Risk evaluation is no longer just a qualitative classification, but directly obtains the loss probability and loss value in a disaster situation. Landslide disaster risk evaluation should first delineate the scope of landslide disaster, and then complete the landslide disaster evaluation, vulnerability evaluation of disaster-bearing body, and the calculation of the value of disaster-bearing body and the total risk value within the scope. In summary, the established landslide disaster risk assessment framework was shown in Fig. 5.

## Delineation method of landslide disaster range based on numerical simulation

For open-pit mines risk evaluation, we should not only care about whether the slope will be unstable, but also care about the influence range and loss of landslide instability. Delineation of landslide disaster area is the basis of landslide risk evaluation. The analysis of landslide movement process is very important. It is necessary to accurately predict the large deformation process of landslide and obtain the parameters characterizing the failure potential of landslide, so as to be used for quantitative risk evaluation of single landslide. With the development of numerical simulation of landslide movement process, the fine simulation of landslide movement process can be realized, and the parameters required for risk evaluation can be directly output. At present, the landslide scope and slip distance prediction methods have been established, which are mainly divided into empirical formula, numerical simulation, mechanical model and statistical regression model. Among them, empirical formula and mechanical model methods are mainly based on the mechanical balance of rock mass or the law of conservation of energy to establish a prediction model^16,17^, and predict the landslide extent by calculating the model. The numerical simulation method has been developed rapidly in recent years, and the more commonly used numerical simulation methods include continuum mechanics method and discrete element method. Compared with the continuum mechanics method, the discrete element method can reflect the separation and aggregation effect between particles during landslide movement, and can effectively simulate the large deformation movement of particles. It is a valuable tool for analyzing and understanding the failure mechanism of landslide. Based on the discrete element method, this study used the numerical simulation method to analyze the stability of the open-pit mine slope to explore the landslide mechanism and predict the landslide disaster range.

### Modeling and simulation results analysis

**Figure 6:**
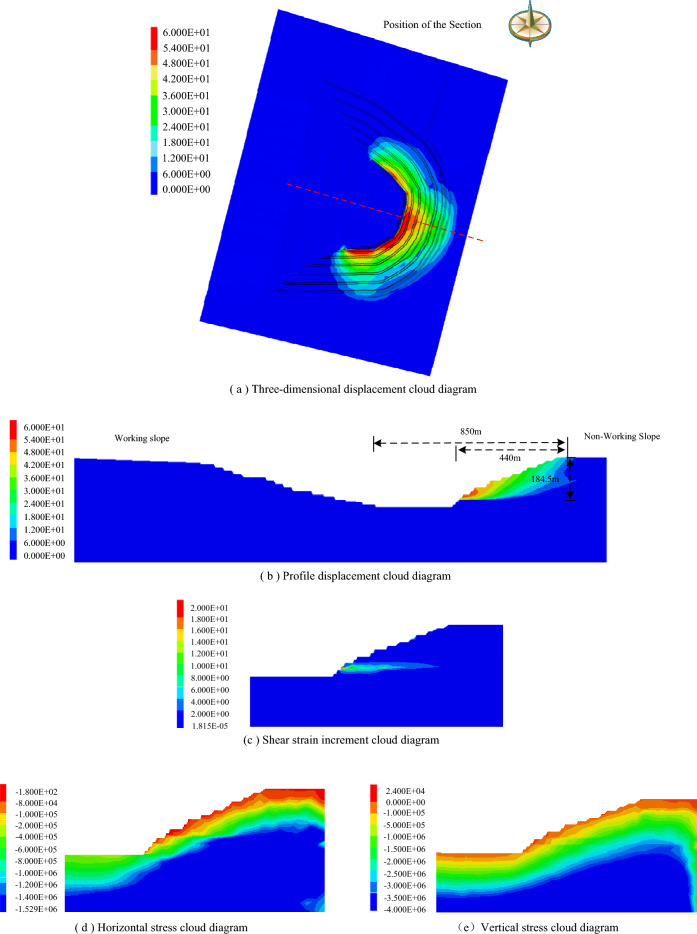
Three-dimensional numerical simulation results.

According to the mining engineering development plan and engineering geological conditions, Flac^3D^ software was used to establish the three-dimensional geological model of the mining field from the east side to the boundaryin the first mining area, including the east side, the north side, the south side and the working side. Through simulation calculation, the most dangerous sliding body shape could be obtained during the period from the first mining area to the boundary. From the analysis of the total displacement cloud diagram (Fig. 6a) and the profile displacement cloud diagram (Fig. 6b), it could be seen that the potential sliding body was located in the east side of the first mining area. The height of the sliding body was about 184.5 m, the width of the sliding body was about 850 m, and the landslide mode was the cutting-bedding sliding with the weak layer of the coal roof as the bottom interface. Figure 6c was the shear strain increment diagram of the east slope of the first mining area, and the failure of the soil-rock slope was mainly caused by the shear strain. The mudstone interlayer at the roof of C1 coal was softened, and the increment of shear strain was obvious. In the interior of the slope, under the action of its own gravity, occurred shear failure and formed a slip surface. The failure body slides out from the roof of C1 coal seam, which was a combined failure in the form of 'cut layer-bedding'. Figure 6d was the horizontal stress cloud diagram. It could be seen from the diagram that the horizontal stress was small on the slope surface and gradually increased to the inside of the slope. However, due to the different rock and soil mass of the slope body had its own mechanical properties. The internal stress distribution was not uniform and changed with the rock layer, which indicated that the internal stress redistribution of the slope body had not reached equilibrium, and the C1 coal roof was the maximum stress position of the slope surface. These were not conducive to slope stability. Figure 6e was a vertical stress cloud diagram. From the slope surface to the bottom, with the increase of buried depth, the vertical stress gradually increased, and the distribution was uniform from top to bottom, and there was no stress concentration. This showed that the vertical direction of the slope was mainly affected by its own gravity.

### Calculation of slip distance

For the determination of the landslide disaster range of this open pit mine, the theoretical maximum slip distance of the landslide body could be determined by selecting the empirical formula comprehensive according to the specific conditions of the landslide body, and then the actual maximum slip distance of the landslide body could be finally determined according to the terrain situation in front of the landslide body. Through the investigation and analysis of the boundary conditions of the landslide lateral edge and the scale characteristics of the landslide body, the influence range on both sides of the landslide lateral edge could be roughly determined, and the potential disaster range of the landslide could be delineated by combining the actual maximum slip distance of the landslide body^18^. In this paper, the slip distance of the landslide could be calculated by using the Sen Xie · Kuan formula and the simple empirical formula summarized by Deng et al.^19^. As follows formulas (1) and (2), and the maximum value was taken as the maximum slip distance after the overall instability of the open-pit landslide. The height (H) of the sliding body was 184.5 m by numerical siulation, and substituted into the formula, the sliding distance of the landslide was about 769 m and 418 m respectively. Through the analysis of the above calculation results, the distance between the front and rear edges of the landslide was 440 m, and the distance from the rear edge of the landslide to the bottom of the working slope was 850 m. The slip distance calculated by the simple empirical formula was 418 m, less than 440 m, so it did not meet the actual conditions. The result calculated by the Sen Xie· Kuan formula was 769 m, greater than 440 m and less than 850 m; The maximum displacement was 60 m by numerical simulation, and the slip distance of landslide was 500 m by calculation. Through comparative analysis, the slip distance of landslide was determined to be 769 m.

① Using the formula of Sen Xie Kuan to calculate

1 $$ \frac{H}{L} = 0.73\tan \varphi - 0.07\quad {\text{namely:}}\;L = \frac{H}{0.73\tan \varphi - 0.07} = \frac{184.5}{{0.73\tan 23 - 0.07}} = 769.177{\text{m}} $$

In the formula: *φ*—the slope angle of the source area (°); *H*—height of sliding body (m); *L*—landslide slip distance (m)*.*

**Figure 7:**
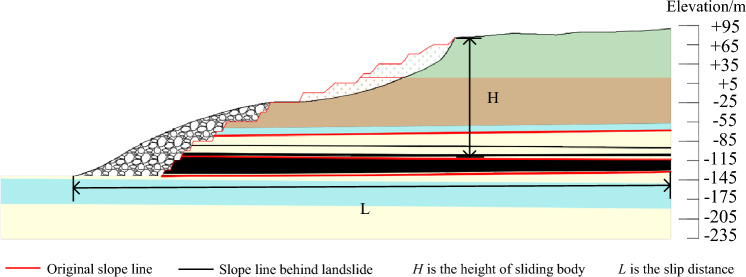
Slip distance diagram.

The sliding distance of the landslide was shown in Fig. 7.

② Using simple empirical formula to calculate

2 $$ L = k\Delta H{ + }B{ = }2.54 \times 184.5{ - }50.378 \approx 418{\text{m}} $$

In the formula: *k*, *B—*coefficient, *k* = 2.0 ~ 2.5, *B* = − 50.378; *H*—height of sliding body (m); *L*—landslide slip distance (m)*.*

### Delineation of landslide disaster range

**Figure 8:**
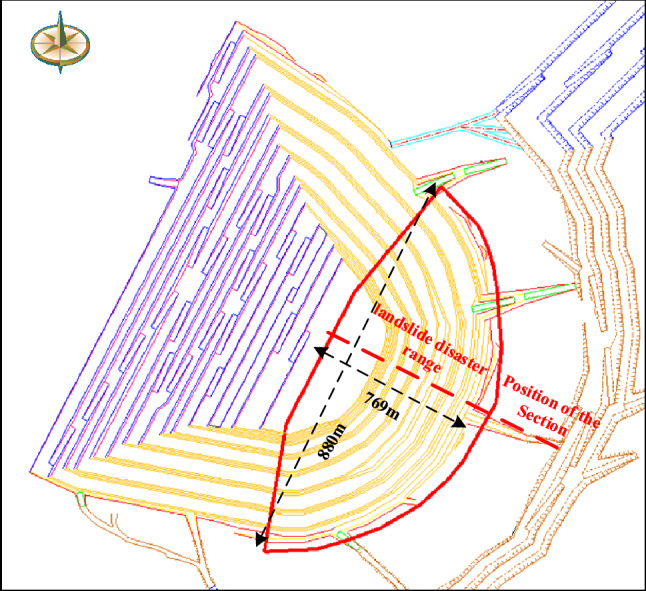
disaster range of landslide.

The scope of landslide disaster refers to the scope of potential landslide damage and the scope of its impact. According to the numerical simulation results, the size of the landslide body and the boundary conditions of the side edge of the landslide body were determined. Combined with the maximum slip distance calculated by theoretical analysis, the potential disaster range of the open-pit mine landslide was delineated, that was an approximate fan shape, and the north–south length was about 880 m and the east–west length was about 769 m. As shown in Fig. 8.

This method can accurately determine the disaster range of open-pit mine landslide, which is more accurate and reliable than the method of calculating the maximum slip distance based on empirical formula and the determined the boundary conditions of landslide side edge through investigation and analysis^20^. Using the powerful data processing, spatial analysis and statistical functions of ArcGIS method, the risk evaluation, vulnerability evaluation, risk evaluation and zoning of landslide disasters in the study area can be completed^21,22^, but ArcGIS method can not accurately delineate the scope of landslide disasters. Comparing the ArcGIS method with this method, the ArcGIS method is more suitable for the evaluation of landslide disasters in a wider area, and the landslide in the open-pit mine is a typical physical risk caused by human activities. The landslide range is relatively small, so it is more necessary to accurately predict the landslide disaster range. The method of numerical simulation combined with theoretical calculation adopted in this paper provides a new idea for the delineation of landslide disaster range, and also provides a certain reference for landslide risk evaluation of other open-pit mines.

## Case analysis of landslide risk quantitative evaluation

### Slope risk evaluation

#### Calculation of landslide instability probability

The typical calculation section was selected within the delineated landslide range. And the two-dimensional slope stability calculation model was established. Based on Monte Carlo method^23^, the probability of landslide instability was calculated by applying Geo-Slope software to the slopes in the study mine under natural conditions, rainstorm conditions and earthquake conditions, respectively.

(1) Natural condition.

**Figure 9:**
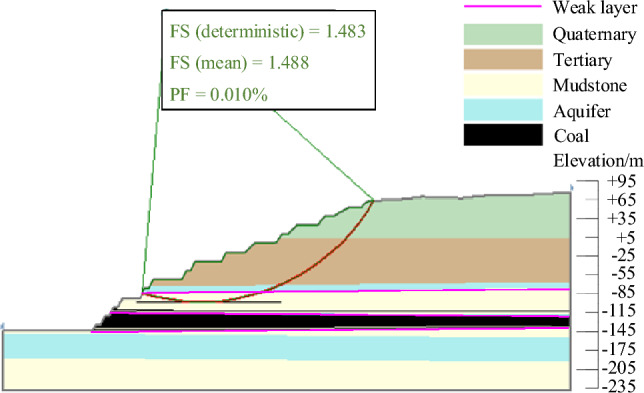
Landslide instability probability of No. 1 sliding surface.

**Figure 10:**
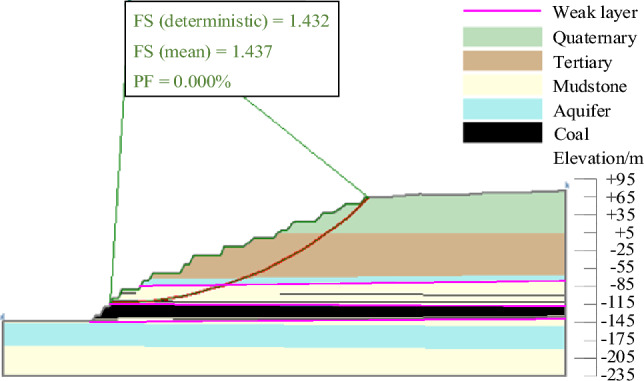
Landslide instability probability of No. 2 sliding surface.

**Figure 11:**
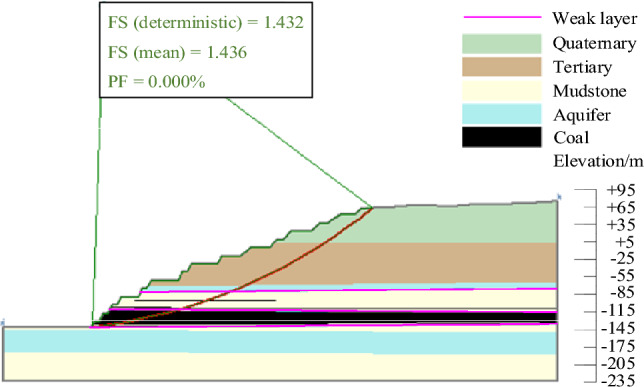
Landslide instability probability of No. 3 sliding surface.

**Figure 12:**
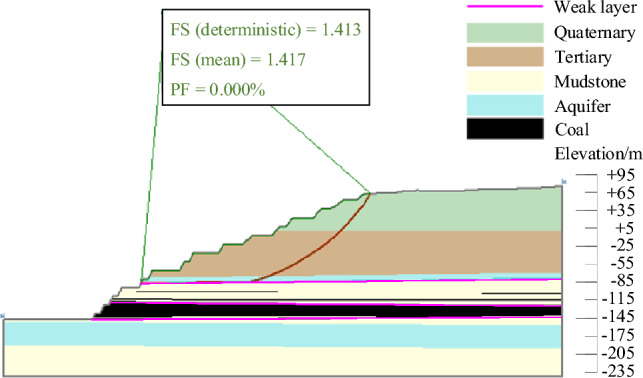
Landslide instability probability of No. 4 sliding surface.

**Figure 13:**
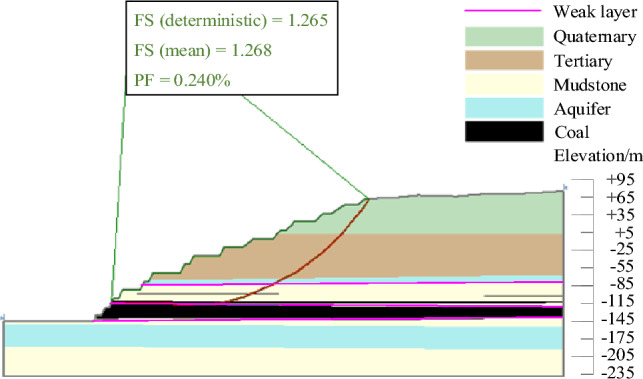
Landslide instability probability of No. 5 sliding surface.

**Figure 14:**
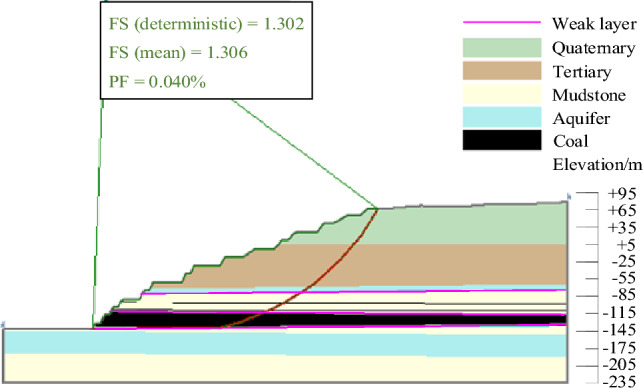
Landslide instability probability of No. 6 sliding surface.

The results of landslide instability probability calculation for six landslide modes of slopes in the study area under natural working conditions were shown in Figs. 9, 10, 11, 12, 13, 14. From the calculation results, it could be seen that the most dangerous slip surface under natural working condition was No.5 slip surface, that was, the sliding surface of cutting-bedding sliding with the weak layer of C1 coal seam roof as the bottom interface. The corresponding maximum instability probability PF of landslide was 0.0024 and the stability coefficient FS(mean) was 1.268.

(2) Rainstorm condition.

**Figure 15:**
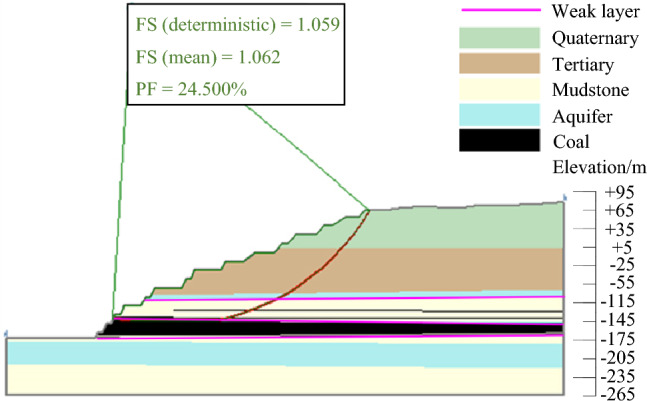
Landslide instability probability under rainstorm condition.

When encountering rainstorm, since the Quaternary floor was aquiclude, the rock layer mainly affected by rainfall was the Quaternary sandstone layer. According to the saturated state of the Quaternary rock mass, the landslide instability probability PF corresponding to the most dangerous sliding surface in the rainstorm state was calculated to be 0.245, and the stability coefficient FS(mean) was 1.062. The results were shown in Fig. 15.

(3) Earthquake condition.

**Figure 16:**
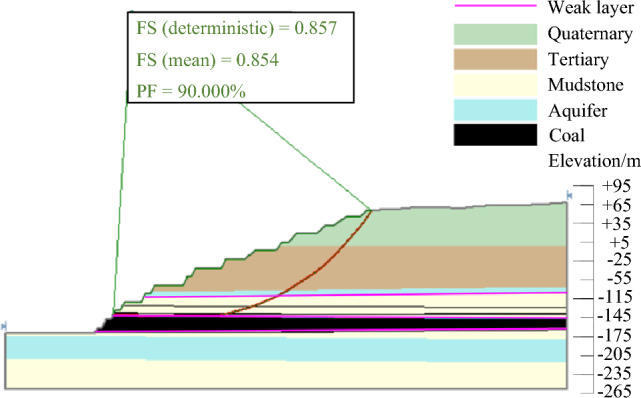
Landslide instability probability under earthquake condition.

According to the survey data, there was a seismic risk in the open-pit mine. The peak acceleration of the earthquake in the Tar area was 0.16–0.24 g, and the basic intensity of the earthquake was VIII. Therefore, the landslide instability probability under the earthquake condition was calculated by slide. As shown in Fig. 16, the instability probability PF of the most dangerous sliding surface under the earthquake condition was 0.9, and the stability coefficient FS(mean) was 0.854.

#### Calculation of annual probability of landslide instability

① Annual probability of landslide instability under natural conditions.

Li and Wu^24^ established the relationship between the annual probability of slope instability during the subsequent service period of slopes with different service years. In this study, when analyzing the annual probability of slope instability under natural conditions of the open-pit mine slope, referring to Li Dianqing 's theory, the annual probability of landslide instability was *P*_*tf*_ = 0.003.

② Annual probability of landslide instability under rainstorm conditions.

Under rainstorm conditions, landslides were prone to occur during extreme rainfall periods. The survey determined that the rainfall return period in the mining area was 10 years, that was, the extreme rainfall probability was *P*_*t*_ = 1/*T* = 0.1. The annual probability of landslide occurrence under rainstorm conditions was calculated as follows: *P*_*tf*_ = *P*_*t*_ × *P*_*f*_ = 0.1 × 0.245 = 0.0245.

③ Annual probability of landslide instability under earthquake conditions.

According to the seismic intensity distribution data of Pakistan, the peak acceleration of the earthquake in the mining area was 0.16–0.24 g, which belonged to the seismic intensity fortification area of VIII. The earthquake recurrence period was *T* = 50 years, and the probability of landslide occurrence was *P*_*t*_ = 1/*T* = 0.02 when the inducing factor was earthquake. According to the determined annual probability of inducing factors, the annual probability of landslide occurrence under earthquake conditions could be calculated as: *P*_*tf*_ = *P*_*t*_ × *P*_*f*_ = 0.02 × 0.9 = 0.018.

Based on the annual probability of landslide instability under different working conditions calculated above, the disaster of the slope was evaluated by semi-quantitative criteria^25^, and the evaluation results were shown in Table 2. The slope of the first mining area of this open pit mine was moderately dangerous under natural conditions. The risk was highly dangerous under seismic conditions and highly dangerous under rainstorm conditions.

**Table 2:** Slope risk evaluation table.

Evaluate working conditions	Annual probability of instability	value ranges	Risk evaluation description
Natural	0.003	1/500–1/100	Middle-risk
Rainstorm	0.0245	1/100–1/20	High-risk
Earthquake	0.018	1/100–1/20	High-risk

### Comprehensive vulnerability evaluation of disaster-bearing body

In quantitative risk evaluation, vulnerability is usually measured by the loss degree or loss value of the disaster-bearing body in the affected area under a certain disaster intensity. The fuzzy comprehensive evaluation-analytic hierarchy process was used to analyze the vulnerability, and the vulnerability evaluation was carried out in combination with the actual situation of the potential landslide impact in the first mining area of the open-pit mine. The four aspects of transportation ramp, house building, mechanical equipment and personnel were used as the criterion layer of the evaluation model to construct the vulnerability evaluation model. Through the analytic hierarchy process, the disaster-bearing body was classified and graded, and the comment set and judgment matrix of the disaster-bearing body are established to calculate the vulnerability of the disaster-bearing body. Finally, the fuzzy evaluation matrix was applied to solve the comprehensive vulnerability of the landslide, which was medium vulnerability^26–29^. According to the quantitative description of vulnerability grade and interval by Xiao^30^, the first mining area of the open-pit mine was evaluated as medium vulnerability, and its value was 0.5. At the same time, by analyzing the types and properties of landslide disaster-bearing bodies, the time probability and spatial probability of each disaster-bearing body within the scope of landslide disaster were obtained, and then the disaster-affected body's disaster-affected probability was 1.

### Value calculation of landslide disaster-bearing body

(1) Value calculation of transport ramp.

The affected transport ramps in the open-pit disaster area were mainly the transport ramps in the pit, and the highways in the disaster area were grade-outside highways. According to the construction prices of different highways, the value of the disaster-bearing body of the transport ramp could be calculated by Formula (3).

3 $$ E_{ri} = \sum\limits_{m = 1}^{n} {E_{rm} } \times L_{rmi} $$

In the formula: *E*_*ri*_—The total economic value of all kinds of highways in unit category i; *E*_*rm*_—Unit value of category m highway; *L*_*rmi*_—The length of category m highway in Unit i.

According to the relevant references^18,31^ and the actual situation, the relevant quota of the construction project in this paper determined that the comprehensive unit price was 500,000 rmb/km. The highway mileage affected by the landslide in the open-pit mine was 1 km, so the value of the transportation ramp in the disaster area was 500,000 rmb.

(2) Value calculation of building facilities.

The structure of building facilities in the disaster area was mainly reinforced concrete structure. According to the construction price of different buildings, the value of disaster-bearing body of building facilities could be calculated by Formula (4).

4 $$ E_{bi} = \sum\limits_{m = 1}^{n} {E_{bm} \times L_{bmi} } $$

In the formula: *E*_*bi*_—The total economic value of all kinds of buildings in unit category i; *E*_*bm*_—Unit value of category m building; *L*_*bmi*_—Total number of category m building in Unit i.

Through investigation, the houses in the landslide disaster-bearing area of the open-pit mine were mainly viewing platforms, maintenance areas and parking lots. The building structure was mainly reinforced concrete structure, with a total building area of about 32,724m^2^. Due to the lack of relevant quotas, this paper took the cost of reinforced concrete structure houses as 1500 rmb/m^2^ according to the valuation unit price of relevant references^18,31,32^, so the value of building facilities in the disaster area was 49,086,000 rmb.

(3) Value calculation of mechanical equipment.

The mechanical equipment in the disaster area was mainly 7 m^3^ excavator, 60 t mining truck, bulldozer, scraper, loader and small hydraulic backhoe. According to the value of different mechanical equipment, the value of the disaster-bearing body of the mechanical equipment could be calculated by Formula (5).

5 $$ E_{ji} = \sum\limits_{m = 1}^{n} {E_{jm} } \times L_{jmi} $$

In the formula: *E*_*ji*_—The total economic value of all kinds of mechanical equipment in unit category i; *E*_*jm—*_Unit value of category m mechanical equipment; *L*_*jmi*_—Number of category m building in Unit i.

Through investigation, the mechanical equipment in the landslide disaster-bearing range of the open-pit mine was mainly 7 m^3^ excavator, 60 t mining truck, bulldozer, loader, small hydraulic backhoe, a total of 15 sets. According to the value of each mechanical equipment, the total economic value was calculated to 30,311,000 rmb.

(4) Value calculation of personnel.

Referring to the 'personal injury compensation method'^33^ for value, the mine had not experienced a large-scale landslide, so the number of deaths caused by landslides was difficult to predict. The loss rate of population value was the loss rate of life value. Therefore, the mortality rate of population in disasters could be calculated by the following equation^31^.

6 $$ {\text{k}} = \sum\limits_{{{\text{i}} = 1}}^{{\text{n}}} {\left( {N_{{\text{i}}} \times D_{{\text{i}}} } \right)} $$

In the formula: *k*—Death toll; *N*_*i*_—The number of threatened people of different ages; *D*_*i*_—Vulnerability at different ages.

Through investigation, the disaster-bearing personnel of the landslide in the open-pit mine were all coal mine workers, with a number of 15 people. The age of the personnel was mainly concentrated around 20–40 years old. According to the investigation and the current national compensation amount for casualties^33^, the average compensation amount in the concentration range of the number of people was 1,045,000 rmb/person, so the value of the personnel in the disaster area was 15,675,000 rmb.

### Calculation of total risk value of landslide and risk decision-making

The value of the transportation ramp, the value of the building facilities, the value of the mechanical equipment and the value of the personnel in the landslide disaster area were calculated respectively. The value of the transportation ramp in the disaster area was 500,000 rmb, the value of the building facilities was 49,086,000 rmb, the value of the mechanical equipment was 30,311,000 rmb, and the value of the personnel was 15,675,000 rmb, totaling 95,572,000 rmb.

When the landslide risk was expressed as risk value, the total risk value mainly refered to the value of landslide loss, for which the calculation could be done by the formula: risk value = probability of landslide instability × probability of disaster-bearing × vulnerability × value of disaster-bearing body^34^, the total landslide risk values under natural conditions, rainstorm conditions and earthquake conditions were 114,686.4 rmb, 11,707,570 rmb and 43,007,400 rmb respectively.

The risk assessment of landslide disasters is mainly considered from two aspects: property loss and human casualties. For property loss, the evaluation method generally uses the profit-loss ratio and the economic loss value under different probability conditions to determine the property loss, and the risk level can be determined according to the property loss defined in the geological disaster classification and description^35^. Therefore, the risk level of this open pit mine was small geological disaster risk under natural conditions and medium geological disaster risk under rainstorm conditions and earthquake conditions. For personnel losses, the evaluation was mainly based on the annual mortality rate of personnel, which could be calculated by the formula: annual mortality rate = annual probability of landslide instability × number of fatalities in a single landslide^32^. And the predicted annual mortality rates of personnel under natural, rainstorm, and earthquake conditions were 0.0225 person/year, 0.18375 person/year, and 0.135 person/year, respectively. According to the F(landslide frequency)-N(expected number of fatalities) criterion method^36^, which was now the most used criterion for acceptable risk levels, it was known that the annual mortality rate of the slopes in the study area of this open pit mine was greater than 10^–3^ persons/year for the population under all three working conditions. Accordingly, it could be determined that the landslide risk of this open pit mine was unacceptable risk. Therefore, it was necessary to take corresponding prevention and control measures for the slopes in the disaster area and increase the prevention efforts to ensure the safe production of open-pit mines.

## Conclusion

1. According to the characteristics of open-pit mine landslide, the quantitative risk evaluation framework of open-pit mine landslide was proposed. At the same time, the landslide disaster range delineation, landslide disaster evaluation, and vulnerability evaluation of disaster-bearing body within the disaster range, value of disaster-bearing body and total risk quantitative evaluation were realized. The above provided a new idea for quantitative risk evaluation of open-pit landslide.
2. Taking taking the open-pit coal mine coal mine in Block I of Thar coal field in Pakistan as the research background, the landslide failure characteristics were obtained through the numerical simulation analysis of slope deformation, and the location of potential sliding body was determined. A method for delineating the landslide disaster range of open-pit mine by numerical simulation combined with empirical formula was proposed. Based on the Monte Carlo method, the slope landslide instability probabilities under natural, rainstorm and earthquake conditions were calculated to be 0.0024, 0.245 and 0.9, respectively, and the predicted instability year probabilities were 0.003, 0.0245 and 0.018, respectively.
3. According to the actual production situation of the mining area, the fuzzy comprehensive evaluation-analytic hierarchy process was used to analyze the vulnerability, and the comprehensive vulnerability of the landslide was medium vulnerability, which was 0.5. By analyzing the types and properties of landslide disaster-bearing bodies, the time probability and spatial probability of each disaster-bearing body within the scope of landslide disaster were obtained, and the bearing probability of the disaster-bearing body was 1. According to the number and value of the disaster-bearing body within the landslide disaster range, the total value of the disaster-bearing body was 95,571,948 rmb, and the total landslide risk value under natural conditions, rainstorm conditions, and earthquake conditions was solved. The values were 114,686.4 rmb, 11,707,570 rmb, and 43,007,400 rmb, respectively.
4. The risk level of economic loss and personnel loss of landslide bearing body was evaluated. The personnel loss was characterized by annual mortality rate, and its risk level exceeded the acceptable risk level. The economic loss was evaluated by the risk level, and the analysis showed that it belonged to the medium-sized geological disaster risk under the conditions of rainstorm and earthquake. Therefore, it was recommended that the open-pit mine in Thar Coalfield, Pakistan needed to take certain landslide prevention and control measures.

## Data Availability

The data used to support the findings of this study are available from the corresponding author upon request.

## References

[1] Cheng Z (2016). Risk assessment of Gantianba landslide in Ningnan County.

[2] Liu H, Yang G, Ye W, Pang L, Shen Y, Wang L (2016). Risk assessment of multi-stage loess landslides based on Monte-Carlo method. Coalf. Geol. Explor..

[3] Luo S (2021). Study on Slope Disaster Risk Assessment of Highway Spoil Ground in Guizhou Mountainous Area.

[4] Mei W, Gu S, Liu X (2022). A quantitative risk assessment method for landslides based on the process of large deformation of landslides. J. Wuhan Univ. (Eng. Edit.).

[5] Zhang L (2002). Study on landslide geological disaster risk assessment based on remote sensing and GIS. Sci. Technol. Inf..

[6] Li Y, Yuan J, Jiang D (2021). GIS-based evaluation of landslide disaster in high mountain valley area–Lushui city as an example. Soil Water Conserv. Res..

[7] Huang J, Lyamin AV, Griffiths DV, Krabbenhoft K, Sloan SW (2013). Quantitative riskassessment of landslide by limit analysis and random fields. Comput. Geotech..

[8] Xiao T, Li DQ, Cao ZJ, Au SK, Phoon KK (2016). Three-dimensional slope reliability and risk assessment using auxiliary random finite element method. Comput. Geotech..

[9] Huang J, Lyamin AV, Griffiths DV (2013). Quantitative risk assessment of landslide by limit analysis and random fields. Comput. Geotech..

[10] Ali A, Huang JS, Lyamin AV (2014). Simplified quantitative risk assessment of rainfall-induced landslides modelled by infinite slopes. Eng. Geol..

[11] Li DQ, Yang ZY, Cao ZJ (2019). Area failure probability method for slope system failure risk assessment. Comput. Geotech..

[12] Li M, Li W, Shi M (2022). Stability evaluation and causal mechanism analysis of Guanyin Mountain landslide based on stochastic theory. World Geol..

[13] Lin F (2023). Simulation analysis of the probability of instability of large-scale landslide accumulation body in mining areas based on mathematical morphology. Energy Environ. Prot..

[14] Peng L, Xu S, Hou J (2015). Quantitative risk analysis for landslides: The case of the Three Gorges area, China. Landslides.

[15] Cao L, Huo L (2011). Stability analysis and prevention of soft rock slope along dip. Tech. Rep..

[16] Long J (2008). Research on Prediction Method of High-Speed Remote Loess Landslide.

[17] Lo CM, Lin ML, Tang CL (2011). A kinematic model of the Hsiaolin landslide calibrated to the morphology of the landslide deposit. Eng. Geol..

[18] Li, B., *Study on risk assessment of Suoertou Landslide in Zhouqu County, Gansu Province*. [Master's thesis]. Beijing: Master's Thesis of China University of Geosciences (2012).

[19] Deng J (2008). Three-Dimensional Dynamic Simulation and Application of Landslide Motion Based on FVM.

[20] Yang Y (2014). Landslide Disaster Risk Assessment Method and its Application.

[21] Wang L, Chang M, Xing Y (2021). Risk assessment of landslide geological disasters based on information method model and GIS. Geol. Disasters Environ. Prot..

[22] Tang F, Lu J, Yuan Y (2018). Risk assessment of landslide disaster caused by mining in loess mining area. Coal Technol..

[23] Li K, Ju N (2014). Slope reliability evaluation based on Monte Carlo method. Chin. J. Geol. Disasters Prevent..

[24] Li D, Wu S (2006). Risk assessment and management of landslide considering time effect. Geomechanics.

[25] Hungr O (2009). Numerical modelling of the motion of rapid, flow-like landslides for disaster assessment. KSCE J. Civ. Eng..

[26] Wang L (1996). Introduction to Analytic Hierarchy Process.

[27] Qu Y, Zhang X (1988). Principle and Application of Fuzzy Mathematics.

[28] Zadeh LA (1978). Fuzzy sets as a basis for a theory of possibility. Fuzzy Sets Syst..

[29] Feng, Z., *et al*. Multi-level fuzzy decision-making for soft foundation treatment scheme of expressway. *Rock Mech. Eng.* (2002) 2.

[30] Xiao H (2019). Dynamic Evaluation Method and Application of Slope Stability in Small and Medium-Sized Open-Pit Mines.

[31] Yin K, Zhang G (2010). Landslide Disaster Risk Analysis.

[32] Xin, L. *Research on Risk Assessment Method of Monolithic Landslide Disaster*. [Master's thesis]. Beijing: Master's Thesis of China University of Geosciences (2008).

[33] Pang M (2005). New exploration on the calculation method and theoretical basis of personal injury compensation. Chin. J. Saf. Sci..

[34] Australian Geomechanics Society. Sub-Committee on Landslide Risk Management (2002). Landslide risk management concepts and guidelines. Aust. Geomech. News J. Aust. Geomech. Soc..

[35] China Geological Disaster Prevention and Control Engineering Industry Association, T/CAGHP 018-2016.

[36] Yuxue Z (1993). Reliability Analysis of Slopes.

